# The Gut Microbiome Correlated to Chemotherapy Efficacy in Diffuse Large B-Cell Lymphoma Patients

**DOI:** 10.3390/hematolrep16010007

**Published:** 2024-01-22

**Authors:** Zhuo-Fan Xu, Li Yuan, Yan Zhang, Wei Zhang, Chong Wei, Wei Wang, Danqing Zhao, Daobin Zhou, Jingnan Li

**Affiliations:** 1 Department of Hematology, Peking Union Medical College Hospital, Peking Union Medical College, Chinese Academy of Medical Science, Beijing 100005, China; xzf16@mails.tsinghua.edu.cn (Z.-F.X.);; 2 School of Medicine, Tsinghua University, Beijing 100084, China; 3 State Key Laboratory of Complex Severe and Rare Diseases, Peking Union Medical College Hospital, Peking Union Medical College, Chinese Academy of Medical Science, Beijing 100005, China; 4 Department of Gastroenterology, Peking Union Medical College Hospital, Peking Union Medical College, Chinese Academy of Medical Science, Beijing 100005, China

**Keywords:** diffuse large B-cell lymphoma, gut microbiota, 16S rRNA, metagenomic sequencing, *Proteobacteria*

## Abstract

The gut microbiome (GMB) has been extensively reported to be associated with the development and prognosis of human diseases. This study aims to investigate the relationship between GMB composition and chemotherapy efficacy in diffuse large B-cell lymphoma (DLBCL). We demonstrated that DLBCL patients at diagnosis have altered GMB compositions. Significant enrichment of the Proteobacteria phylum in DLBCL patients was observed. Gene analysis showed a high abundance of virulence factors genes. We found baseline GMB to be associated with clinical outcomes. The emergence of *Lactobacillus fermentum* was correlated with better treatment outcome. Our pilot results suggested a correlation between GMB composition and DLBCL development and prognosis. Clues from our study, together with previous research, provided a rational foundation for further investigation on the pathogenesis, prognosis value, and targeted therapy of GMB in DLBCL.

## 1. Introduction

In recent years, preclinical and clinical studies have demonstrated the correlation between the gut microbiome (GMB) and several human diseases, including metabolic disorders, autoimmune diseases, neurologic conditions, and cancers [1,2,3,4,5,6,7]. Several studies have also found associations between GMB composition and the response to cancer therapy, including chemotherapy [8,9], immune checkpoint blockade [10,11,12], and allogeneic hematopoietic cell transplantation [13]. The underlying mechanisms are incompletely understood but include nutrient processing, polysaccharide synthesis, and immunomodulating [14,15]. These studies have spurred interest in other diseases with unclear etiology and pathogenesis where a connection to the microbiome is suspected.

Hematologic malignancies have an intricate relationship with host immunity and a long history of treatment with immune-based therapies. Therefore, they provide a unique opportunity to understand how GMB may influence the relationship between the host, disease, and therapy. The identification of the association between *Helicobacter pylori* and gastric mucosa-associated lymphoid tissue lymphoma serves as a quintessential example of how GMB can directly promote the development of cancers [16]. In leukemia, it was demonstrated that GMB populations can distinguish patients at diagnosis [17]. In B-cell non-Hodgkin lymphoma, recent research has shown that microbial dysbiosis is associated with aggressive histology and adverse clinical outcome [18].

Diffuse large B-cell lymphoma (DLBCL) is the most common non-Hodgkin lymphoma. It accounts for over 60% of non-Hodgkin lymphoma cases in China. Overall, DLBCL is an aggressive but potentially curable malignancy. The majority of patients can achieve complete remission with standard chemotherapy with rituximab. However, there are still 40% of patients who do not benefit from the current standard treatment. Despite its high prevalence, the etiology and pathogenesis of DLBCL are not fully disclosed. To address this challenge, more studies are needed in order to uncover the mechanisms of DLBCL development, discover predictors for treatment response, and expand the treatment options. Previous research has shown the association between DLBCL and GMB composition [18,19,20]. Here, we investigated the relationship between GMB composition and specific taxon abundance with DLBCL development and clinical outcome.

## 2. Materials and Methods

### 2.1. Sample Collection of Patients and Healthy Controls

The DLBCL patients (*n* = 17) were recruited in the Hematology Department of Peking Union Medical College Hospital (PUMCH) from November 2019 to November 2020. They were diagnosed as DLBCL according to pathological biopsy of lymph nodes [21]. The exclusion criteria included the following: chemotherapy, antibiotic treatment within 4 weeks, chronic gastrointestinal inflammatory diseases, other tumors, history of indolent lymphoma, active viral infection, and history of autoimmune disease. Fecal samples of the patients were collected before (pre-treatment group, PRG) and after 4 courses of chemotherapy (post-treatment group, POG). The post-treatment time point of the fecal collection was set when the blood routine returned to normal. The healthy controls (*n* = 18) consisted of employees at PUMCH who consented to the collection of a fecal sample used for microbiome analysis. They were matched with the DLBCL patients for age and sex. The exclusion criteria for healthy controls were as follows: active infection within 4 weeks, antibiotic treatment within 4 weeks, history of autoimmune diseases and tumor.

### 2.2. Treatment and Response

All patients received R-CHOP (rituximab 375 mg/m^2^ iv d0, cyclophosphamide 750 mg/m^2^ iv d1, epirubicin 75 mg/m^2^ iv d1, vindesine 4 mg iv d1, prednisone100 mg po d1-5) therapy. The response was evaluated via positron emission tomography/computed tomography, and remission was determined based on the Deauville five-point method after four courses of chemotherapy. Patients with a score under 3 points are defined as complete remission (CR), while above 4 points are defined as non-complete remission (NCR). The grouping information was demonstrated in Figure S1. The PET/CT images of patients with CR/NCR were demonstrated in Figure S2.

### 2.3. 16S rRNA Sequencing

The microbiota composition of fecal samples from 17 DLBCL patients (including 17 PRG samples and 17 POG samples) and 18 healthy controls were analyzed by 16S ribosomal RNA gene sequencing. The fresh feces were collected aseptically in the fecal collection tube (MicroLocker). Immediately after sampling, the specimens were stored in a −20 °C freezer. Within 24 h, they were transferred to a −80 °C freezer until DNA was extracted.

Fecal DNA was extracted using PowerSoil DNA Isolation Kit (MoBio Laboratories, Carlsbad, CA, USA) following the recommended protocol. The purity and quantity of the genomic DNA were checked on 1% agarose gels and a NanoDrop spectrophotometer (Thermo Scientific, Waltham, MA, USA), respectively. The V3-4 hypervariable regions of bacterial 16S rRNA gene were amplified with the primers 338F (ACTCCTACGGGAGGCAGCAG) and 806R (GGACTACHVGGGTWTCTAAT) [15]. The PCR products were purified using an Agencourt AMPure XP Kit. Deep sequencing was performed on Miseq platform at Allwegene Company (Beijing, China). After the run, image analysis, base calling, and error estimation were performed using Illumina Analysis Pipeline Version 2.6. The original sequence was uploaded to the SRA database of NCBI. The raw data were first screened, and sequences were removed from consideration if they were shorter than 230 bp, had a low quality score (≤20), contained ambiguous bases, or did not exactly match primer sequences and barcode tags. Qualified reads were separated using the sample-specific barcode sequences and trimmed with Illumina Analysis Pipeline Version 2.6, and then the dataset was analyzed using QIIME. The sequences were clustered into operational taxonomic units (OTUs) at a similarity level of 97% [16] to generate rarefaction curves and to calculate the richness and diversity indices. The Ribosomal Database Project (RDP) Classifier tool was used to classify all sequences into different taxonomic groups [17]. QIIME1 (v1.8.0) software was used to analyze the α-diversity index (including Shannon, Simpson, and Chao1 indexes). Partial least squares discrimination analysis (PLS-DA) was used to analyze β-diversity using R (v3.6.0). The differences between groups were analyzed by using the software of mothur (v.1.34.4 [18]), and LEfSe was analyzed by python (V2.7).

### 2.4. Metagenomic Sequencing

Metagenomic sequencing were performed in all 52 samples. The purified DNA was sheared to 300 bp with the Covaris ultrasonic crusher. To prepare the sequencing library, the fragments were treated by end repair, A tailing, and ligation of Illumina-compatible adapters. DNA sequencing libraries were deep-sequenced on the Illumina Hiseq platform at Allwegene Company (Beijing, China). After the run, image analysis, base calling, and error estimation were performed using Illumina Analysis Pipeline Version 2.6. The quality control of the raw data was carried out by using Trimmomatic [22], including the removal of adapter sequences and low-quality reads. We filtered the reads if they were with the adapter sequence, N (uncertain base) ratio greater than 1%, and the content of low-quality base (Q ≤ 20) greater than 50%, and we filtered out the reads whose length was still less than 150 bp after quality control. High-quality sequences were compared with NR database and classified into different taxonomic groups by using DIAMOND [23] tool. MEGAHIT [24] was used to assemble the sequencing data, and the contigs below 500 bp were filtered out. Contigs were annotated with Prodigal software (V2.6.3) to predict open reading frames (ORFs) [25], and CD-HIT software (http://cd-hit.org/) [26] was used to construct the non-redundant gene set. Bowtie [27] was used to compare the sequencing data with the non-redundant gene set, and the abundance information of genes in different samples was counted. The gene function was annotated by searching against the functional annotation database KEGG, COG/KOG, GO, CARD, and CAZyme.

## 3. Results

### 3.1. Patients Characteristics

This prospective study recruited 17 untreated DLBCL patients (PRG) and 18 matched healthy control (control group, CG). The PRG included eight males (47.1%) and nine females (52.9%). The 17 DLBCL patients included GCB (9 cases, 52.9%) and ABC (8 cases, 47.1%) pathological subtypes. There were 6 patients with gastrointestinal involvement (GI, 35.3%) and 11 patients without (NGI, 65.7%). IPI scores were assessed for all patients at diagnosis. All patients were treated with the R-CHOP-based regimens. During treatment, 13 patients (76.4%) received antibiotics due to febrile neutropenia. Response rates were assessed after four courses of chemotherapy; 10 patients (58.5%) reached metabolic CR and 7 patients (41.2%) were NCR. The median age of the CG was 53 years old (range: 24–74 years), including 10 males (55.6%) and 8 females (44.4%). There was no significant difference in age and gender between the PRG and the CG (*p* = 0.50 and 0.81, respectively). The baseline characteristics of the cohort are listed in Table 1.

### 3.2. GMB Composition Was Altered in DLBCL Patients

**Figure 1:**
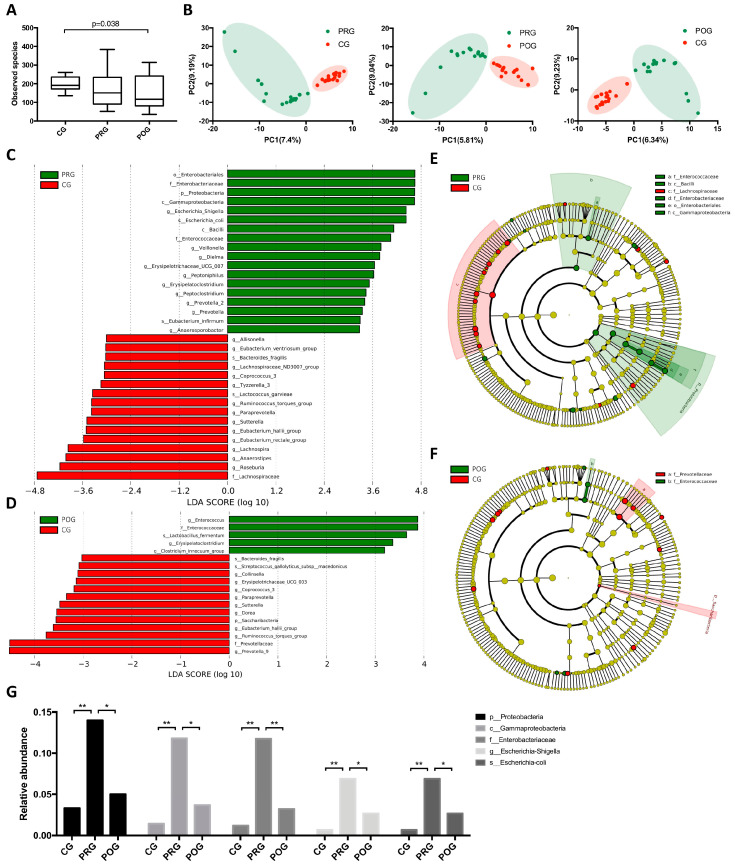
GMB composition of healthy controls, untreated patients, and treated patients. (CG: control group; PRG: pre-treatment group; POG: post-treatment group.) (**A**) α-diversity analysis of CG, PRG, and POG. The observed species was significantly decreased in the POG compared to CG (*p* = 0.038). (**B**) β-diversity by PLS-DA analysis. The GMB composition of CG, PRG, and POG differed from each other. (**C**) LEfSe analysis of taxa abundance, PRG vs. CG. (**D**) LEfSe analysis of taxa abundance, POG vs. CG. (**E**) Cladogram, PRG vs. CG. (**F**): Cladogram, POG vs. CG. (**G**) The relative abundance of *Proteobacteria* phylum in CG, PRG, POG. * indicated *p* < 0.05, ** indicated *p* < 0.01.

To investigate whether GMB was altered in untreated DLBCL patients and how GMB composition changed throughout chemotherapy, we prospectively collected fecal samples from DLBCL patients at pre-treatment and post-treatment timepoint (PRG, *n* = 17; POG, *n* = 17), as well as from healthy controls (CG, *n* = 18). A total of 52 fecal specimens were analyzed by 16S rRNA gene sequencing and metagenomic sequencing. By α-diversity analysis, no significant difference was observed between PRG and CG. However, there was a larger within-group variation in PRG compared to CG. When POG was compared to CG, a significant decrease in GMB diversity was observed (Figure 1A and Figure S4). These results indicated the potential correlation of GMB diversity with the disease state and treatment. We then analyzed whether GMB composition was altered in DLBCL patients. β-diversity analysis revealed significant differences in GBM composition among the three groups (Figure 1B). Taxonomy analysis was performed using linear discriminant analysis (LDA) of effect size (LEfSe) to characterize taxa differences. By comparing PRG to CG, we found a higher abundance of phylum *Proteobacteria* (*p* = 0.019, Kruskal–Wallis test), including the class *Gammaproteobacteria* (*p* = 0.0078), order *Enterobacteriales* (*p* = 0.0052), family *Enterobacteriaceae* (*p* = 0.005), genus *Escherichia-Shigella* (*p* = 0.008), and species *Escherichia coli* (*p* = 0.008) in PRG. The other taxon that was enriched in DLBCL patients was phylum *Enterobacteriaceae*, including the class *Bacilli* and family *Enterococcaceae* (Figure 1C,E). To further investigate whether the abundance of phylum *Proteobacteria* and *Enterobacteriaceae* changed after chemotherapy, we compared the taxa difference between PRG and POG, POG and CG. As shown, the dominance of *Proteobacteria* was restored after treatment. The abundance of *Enterobacteriaceae* was significantly reduced in POG compared to PRG; however, it remained high in POG compared to CG (Figure 1D,F and Figure S3). The alteration of the relative abundance of *Proteobacteria* phylum throughout treatment is demonstrated in Figure 1G. These results suggested a potential role of *Proteobacteria* in the development of DLBCL. In addition, we also compared the GMB diversity and composition differences between the GCB (*n* = 9) and ABC (*n* = 8) subtypes of DLBCL. However, we did not find significant differences between the two pathologic subtypes.

**Figure 2:**
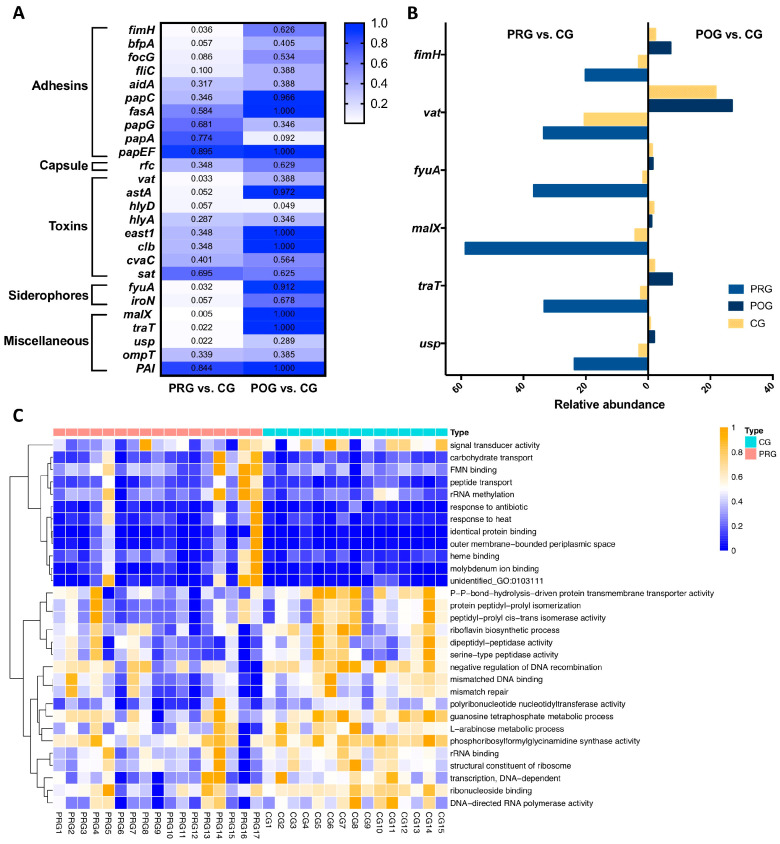
Gene expression of healthy controls, untreated patients, and treated patients. (CG: control group; PRG: pre-treatment group; POG: post-treatment group.) (**A**) Heatmap with *p* values (Kruskal–Wallis test) for differences in virulence gene abundance between groups. (**B**) The relative abundance of genes that reached significance (*p* < 0.05) in Kruskal–Wallis tests. (**C**) Pathway analysis (annotated to GO database), PRG vs. CG.

To further elucidate, we looked into gene level for evidence of *Proteobacteria*’s role in DLBCL via metagenomic sequencing. Targeted analysis was performed on a set of genes of interest. These genes were chosen based on previous reports of their functional relevance to the virulence of *Proteobacteria*, especially *E. coli*, in the context of human diseases [28,29,30]. We compared 26 genes that function as adhesins, capsules, toxin producers, siderophore receptors, outer membrane proteins, etc. Six genes were found to be enriched in DLBCL patients, including *fimH* (an adhesin gene), *vat* (a vacuolating toxin gene), *fyuA* (the yersiniabactin receptor gene), *malX* (the pathogenicity island marker), *traT* (a gene associated with serum survival), and *usp* (an uropathogen-specific gene). After treatment, the abundance of the above genes decreased to the baseline level of CG (Figure 2A,B; Table S1). In the untargeted analysis, we annotated the function of the abundant genes by searching against the functional annotation database GO [31,32]. In PRG, there was decreased activity in pathways involved in peptide digestion, DNA recombination, DNA mismatch repair, rRNA transcription, and the guanosine metabolic process (Figure 2C). Altogether, these results proved the correlation between the Proteobacteria phylum and untreated DLBCL.

### 3.3. GMB Composition Was Associated with Chemotherapy Efficacy

**Figure 3:**
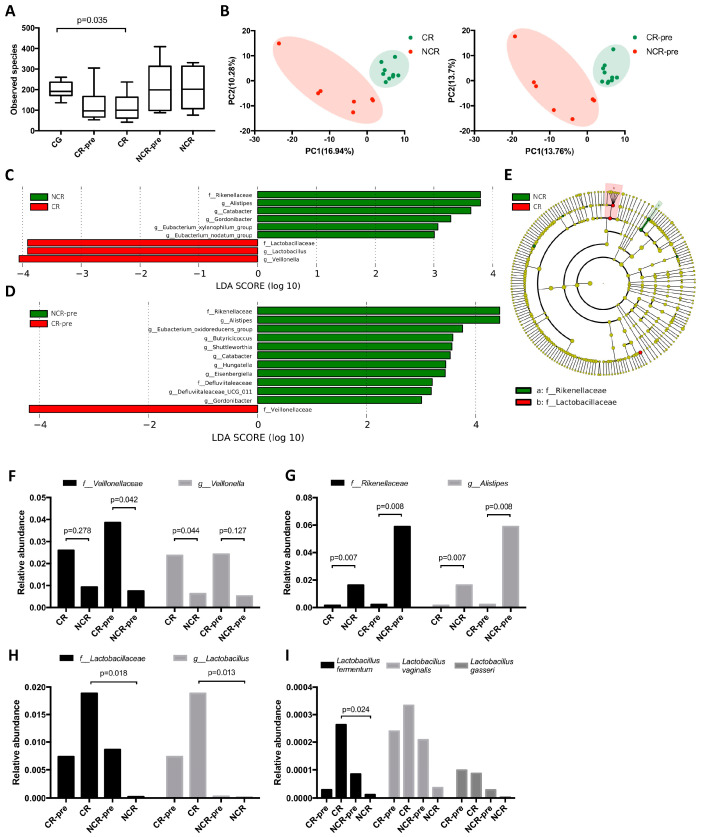
GMB composition of CR, NCR, CR-pre, and NCR-pre patients. (CR: post-treatment patients with complete remission; NCR: post-treatment patients without complete remission; CR-pre: pre-treatment patients who reached complete remission after chemotherapy; NCR-pre: pre-treatment patients who did not reach complete remission after chemotherapy.) (**A**) α-diversity analysis of each group. The observed species was significantly decreased in the CR compared to CG (*p* = 0.035). (**B**) β-diversity PLS-DA analysis. The GMB composition of each group was different. (**C**) LEfSe analysis of taxa abundance, CR vs. NCR. (**D**) LEfSe analysis of taxa abundance, CR-pre vs. NCR-pre. (**E**) Cladogram of CR vs. NCR. (**F**) The relative abundance of *Veillonellaceae* family in each group. (**G**) The relative abundance of *Rikenellaceae* family in each group. (**H**) The relative abundance of *Lactobacillaceae* family in each group. (**I**) The relative abundance of *Lactobacillus* species in each group.

To investigate how the GMB affects chemotherapy efficacy in DLBCL, we sub-grouped the post-treatment samples into complete remission (CR, *n* = 10), non-complete remission (NCR, *n* = 7), and the pre-treatment samples to complete remission pre-treatment (CR-pre, *n* = 10) and non-complete remission pre-treatment (NCR-pre, *n* = 7). In the α-diversity analysis, a decrease in GMB diversity was observed in CR compared to CG (*p* = 0.035). The differences between CR and NCR did not reach statistical significance (Figure 3A and Figure S4). In the β-diversity analysis, we compared CR to NCR, and CR-pre to NCR-pre. Significant differences in GMB composition were found in CR and NCR patients both before and after treatment (Figure 3B). This indicated the potential role of GMB composition in treatment response. We then performed LEfSe analysis between CR and NCR groups. It was demonstrated that family *Lactobacillaceae*, genus *Lactobacillus*, and genus *Veillonella* had significantly higher abundance in CR patients, while family *Rikenellacea*, genus *Alistipes* were more prevalent in NCR patients (Figure 3C,E). To investigate whether these compositional differences existed before treatment, we compared CR-pre and NCR-pre patients. Consistent predominance was observed for the family *Veillonellaceae* in CR-pre, and family *Rikenellaceae*, genus Alistipes, in NCR-pre (Figure 3D). The relative abundance of *Veillonellaceae* and *Rikenellaceae* in each group were plotted (Figure 3F,G). This made us wonder whether the pre-existence of *Veillonellaceae* or *Rikenellaceae* could serve as a predictor for treatment response. We evaluated the relationship between the relative abundance of *Veillonellaceae/Rikenellaceae* and treatment response using Bayesian logistic regression. We established that high relative abundance (>1%) of *Veillonellaceae* was significantly correlated with decreased risk of NCR (*p* = 0.039, OR = 0.071, 95% CI (0.006, 0.881)). On the contrary, high relative abundance of *Rikenellaceae* (>1%) was associated with increased risk of NCR (*p* = 0.069, OR = 1.022, 95% CI (0.998, 1.045)), while *Rikenellaceae* did not show regression significance. The cut-off value was determined via ROC analysis (Table S3).

In the LEfSe analysis of CR and NCR, we found family *Lactobacillaceae*, genus *Lactobacillus* had significantly higher abundance in CR compared to NCR, but not in CR-pre compared to NCR-pre. This indicated that *Lactobacillaceae* emerged after treatment (Figure 3C,D). We then searched which species of *Lactobacillus* were increased during treatment and found that among the three species being detected in the samples, *Lactobacillus fermentum* was significantly higher in CR compared to NCR (Figure 3H,I).

**Figure 4:**
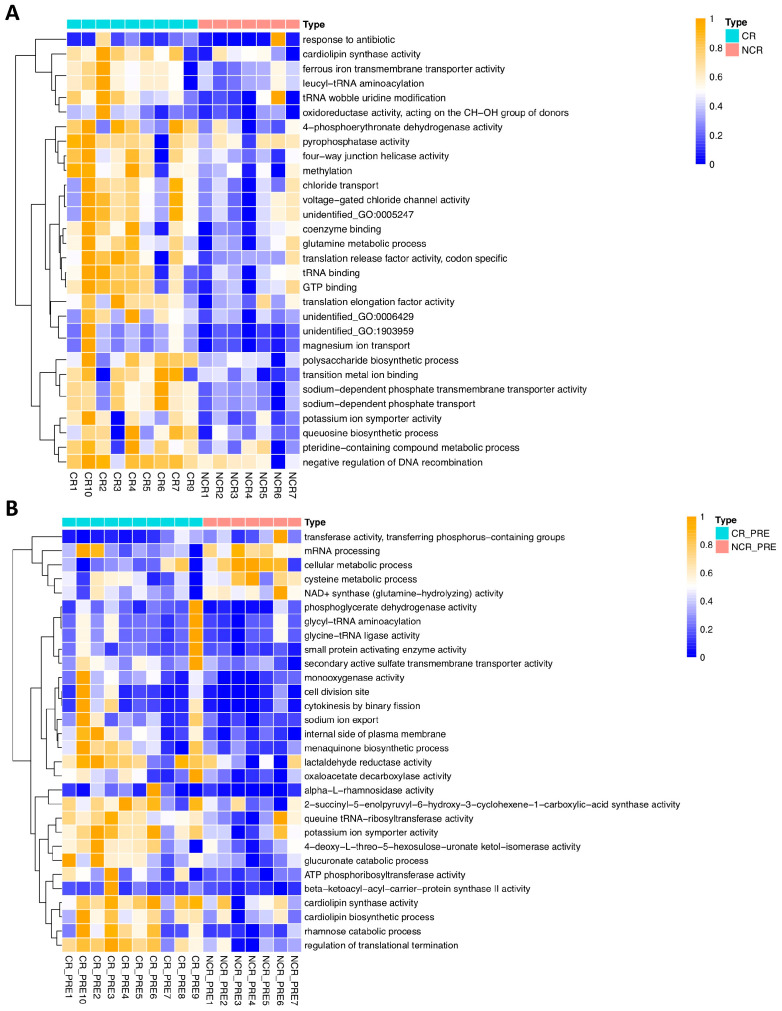
Pathway analysis of CR, NCR, CR-pre, and NCR-pre groups. (CR: complete remission group; NCR: non-complete remission group; CR-pre: complete remission pre-treatment; NCR-pre: non-complete remission pre-treatment). (**A**) CR vs. NCR (annotated to GO database). (**B**) CR-pre vs. NCR-pre (annotated to GO database).

To investigate which pathways were associated with treatment outcomes, we performed untargeted analysis by annotating against the GO database and compared CR to NCR and CR-pre to NCR-pre. We found that cardiolipin synthase activity and potassium ion symporter activity were enriched in both CR and CR-pre groups. Despite these two pathways, other pathways enriched in the CR and CR-pre patients were different, indicating that the treatment response is a complicated process that involved GMB function and gut immune modulation (Figure 4A,B).

The polysaccharide synthesis pathway was enriched in CR patients. In lactic acid bacteria, this pathway has been associated with health-promoting benefits. In particular, the exopolysaccharide (EPS) synthesis pathway has been postulated to play important roles in microbial-mediated immunomodulation [33,34]. Thus, we further investigated genes related to the probiotic characteristics of *L. fermentum* by targeted analysis. We found *epsF*, one of the EPS-related genes, to be significantly higher in CR compared to NCR (Table S2).

We also investigated the correlation of gut microbiome composition (especially *Veillonellaceae*, *Rikenellaceae,* and *Lactobacillaceae*) and host immunology status. We found that patients with higher abundance of *Rikenellaceae* tend to have higher level of inflammation markers (lactate dehydrogenase (LDH) and iterlukin-8) and immunoglobulins (IgG and IgA). When comparing the serum inflammation marker (LDH) before and after treatment, we found patients with higher abundance of *Lactobacillaceae* had a greater decrease in LDH level in response to chemotherapy (Figure S5).

## 4. Discussion

Our study suggested that DLBCL patients at diagnosis have altered GMB compositions. We found a higher abundance of *Proteobacteria* phylum and *Enterobacteriaceae* phylum in DLBCL patients. Gene analysis showed enrichment of several virulence factors genes, which further elucidated the possible effect of *Proteobacteria* in the development of DLBCL. The role of *Proteobacteria*, especially *E. coli* in human diseases, has been extensively reported. Some *E.coli* strains carry virulence genes that enable them to induce cellular modulating or genotoxic effects on host cells [35]. The gene products, called cyclomodulins, were reported to modulate cellular differentiation, apoptosis, and proliferation [36]. For example, colibactin, encoded by the *pks* genomic island causes DNA double-strand breaks and chromosomal instability in eukaryotic cells [30]. DLBCL harbored aberrantly active somatic hypermutation. Studies had identified several driver mutations in the pathogenesis of the disease [37]. The mechanism of these mutations remained largely unknown. Generally, it is considered that GCB and ABC subtypes of DLBCL harbored different gene mutations and developed distinct pathological manifestation. However, our cohort did not show significant differences between the two subtypes. Together with previous findings, our results provided strong rationales to further investigate the pathogenesis role of *Proteobacteria* in DLBCL. Potentially, regulating the abundance of gut *Proteobacteria* phyla may be a novel prevention strategy for the disease.

Furthermore, we illustrated that baseline GMB was associated with clinical outcomes. The relative abundance of the *Veillonellaceae* and *Rikenellaceae* family was significantly different in CR and NCR patients. *Veillonella* are anaerobic Gram-negative cocci and opportunistic pathogenic bacteria. The overabundance of these bacteria has been associated with intestinal inflammation diseases. Studies have shown their immunomodulatory properties by inducing IL-6 and activating macrophages via the LPS-TLR4 pathway [38,39]. *Alistipes* was a relatively new genus of bacteria with emerging implications in human diseases [40]. In terms of pathogenicity, there is contrasting evidence indicating that *Alistipes* may have protective effects against some diseases, including liver fibrosis, colitis, and hepatocellular carcinoma [41,42,43]. In contrast, other studies indicated that *Alistipes* are pathogenic in colorectal cancer by activating the IL-6/STAT3 pathway [44,45]. The tumor microenvironment has recently been increasingly recognized as a biomarker for novel therapy in hematological malignancies. The immune landscapes in DLBCL appear to be heterogeneous and could be modulated by intrinsic molecular or genetic features of the lymphoma cells, but also by other factors, such as the immunological status of the patient. Our study found that patient immunological status, including serum inflammation markers and immunoglobulin levels, were associated with GMB composition. These results provided a rationale to further investigate the immunomodulating effects of GMB on the DLBCL tumor microenvironment.

Finally, we showed that the emergence of *Lactobacillus fermentum* correlated with better treatment response. The abundance of *Lactobacillaceae*, *Lactobacillus*, and *Lactobacillus fermentum* were significantly higher in CR than in NCR, while there were no differences between CR-pre and NCR-pre. These results revealed that the emergence of *L. fermentum* during chemotherapy might be associated with tumor extinction and better treatment response. *Lactobacilli* are subdominant components of the human intestinal microbiota. They are generally considered probiotics that are associated with health benefits, including anticancer properties with respect to the host [46,47]. In colorectal cancer, probiotic bacteria are among the new therapeutic options under evaluation. The underlying mechanism of *Lactobacilli*’s effect on tumor development and growth included the induction of apoptosis, the immunomodulation of inflammatory cytokines, and the changing profile of the gut microbial community [48]. More specifically, *Lactobacilli* have been reported to have antiproliferative effects by regulating apoptosis-regulatory protein BCL2, and to have an anti-metastasis effect by suppressing the VEGF/MMPs signaling pathway [48,49,50,51,52]. In lymphoma, similar protective effects have been reported [53]. The production of exopolysaccharides (EPS) is one of the unique features of *Lactobacillus* genus that contributes to its probiotic effects. We also found *epsF* to be enriched in CR. This indicated that there might be other pathways involved in polysaccharide biosynthesis that fulfills the probiotic function of *Lactobacillus*.

In terms of managing gut microbiota for better disease outcome, common strategies include probiotics, fecal microbiota transplantation, and restraining antibiotics use. In hematological malignancies, it is common for immune-compromised patients to be administered broad-spectrum antibiotics. However, previous studies have found associations among antibiotics use, low gut microbiota diversity, and higher mortality in allogeneic hematopoietic cell transplantation patients [13]. This study suggested that rational interventions to restore the integrity of gut microbiota were potentially beneficial.

In conclusion, our results suggested a possible correlation between GMB composition and DLBCL development and prognosis. However, the impact of these pilot results is limited by our small sample size. The validation of these findings in larger clinical studies will be needed. Moreover, additional microbiome data at different treatment time points such as long-term follow-up will help us to better understand the role of the GMB in DLBCL therapy. Potentially, these findings could have major implications for improving treatment outcomes by identifying response predictors that could facilitate personalized treatment by using easily obtainable fecal samples. Importantly, our findings may inform the development of personalized, microbe-targeted therapies, a new minimally toxic treatment paradigm for aggressive and treatment-refractory lymphomas.

## Figures and Tables

**Table 1:** Characteristics of DLBCL patients and healthy controls.

	Patients (*n* = 17)	Control (*n* = 18)
**Median age, yr**	55	53
**Gender, *n* (%)**		
Male	8 (47.1)	10 (55.6)
Female	9 (52.1)	8 (44.4)
**Race/region, *n***		
East Asian/China	17	18
**Pathological subtype, *n* (%)**		
GCB	9 (52.1)	
ABC	8 (47.1)	
**Organ involved, *n* (%)**		
GI	6 (35.3)	
NGI	11 (64.7)	
IPI * score, *n* (%)		
0	2 (11.8)	
1	5 (29.4)	
2	4 (23.5)	
3	4 (23.5)	
4	1 (5.9)	
5	1 (5.9)	
**Treatment (%)**		
R-CHOP ^+^	17 (100)	
**Response (%)**		
CR	10 (58.5)	
NCR	7 (41.2)	
**Antibiotics use (%)**		
Received	13 (76.4)	
Not received	4 (23.6)	

* International prognostic index. ^+^ Details of chemotherapy were described in materials and methods.

## Data Availability

The data that support the findings of this study are openly available in figshare at http://doi.org/10.6084/m9.figshare.22032593.

## Supplementary Materials

The following supporting information can be downloaded at https://www.mdpi.com/article/10.3390/hematolrep16010007/s1: Figure S1: Schematic outline of the study design. Figure S2: PET/CT images of CR/NCR patients. For each patient, left: pre-treatment; right: post-treatment (4x R-CHOP). Figure S3: GMB composition of untreated patients and treated patients. (UG: untreated patients; TG: treated patients.) A: LEfSe analysis of taxa abundance, UG vs. TG. B: Cladogram, UG vs. TG. Figure S4: A–I: The α-diversity analysis of each pair of groups. The *p*-values by Turkey-group-test were indicated in the figures. Figure S5: A–F: The correlations of serum inflammation markers/immunology markers (LDH, IL-6, IL-8, IgG, IgA, IgM) and relative abundance of *Veillonellaceae*. G–L: The correlations of serum inflammation markers/immunology markers (LDH, IL-6, IL-8, IgG, IgA, IgM) and relative abundance of *Rikenellaceae*. Figure S6: The serum LDH level before and after treatment. Patients with high *Lactobacillaceae* abundance (post-treatment) were labeled with the red color. Table S1: Virulence factors of *E.coli* in PRG vs. CG. Table S2: Exopolysaccharides synthesis pathway in *Lactobacillus* in CR vs. NCR. Table S3: Patients data of GMB relative abundance, treatment response, and serum tests.
