# Supplementary material for: The Gut Microbiome Correlated to Chemotherapy Efficacy in Diffuse Large B-Cell Lymphoma Patients
Source: Hematol Rep. 2024 Jan 22;16(1):63–75. doi: 10.3390/hematolrep16010007 (PMC10885071; doi:10.3390/hematolrep16010007)
Supplement: Supplementary file 1 [file hematolrep-16-00007-s001.zip › Supplementary_Material_DLBCL.pdf]

Supplementary Material

The Gut Microbiome Correlated to Chemotherapy Efficacy in Diffuse Large B-cell Lymphoma Patients

Zhuo-Fan XU[1,4†], Li YUAN[1†], Yan ZHANG[1], Wei ZHANG[1], Chong WEI[1], Wei WANG[1], Danqing ZHAO1, Daobin ZHOU1,3*, Jingnan LI[2]

[*]**Correspondence:** Daobin ZHOU: zhoudb@pumch.cn

1 Supplementary Figures and Tables

1.1 Supplementary Figures

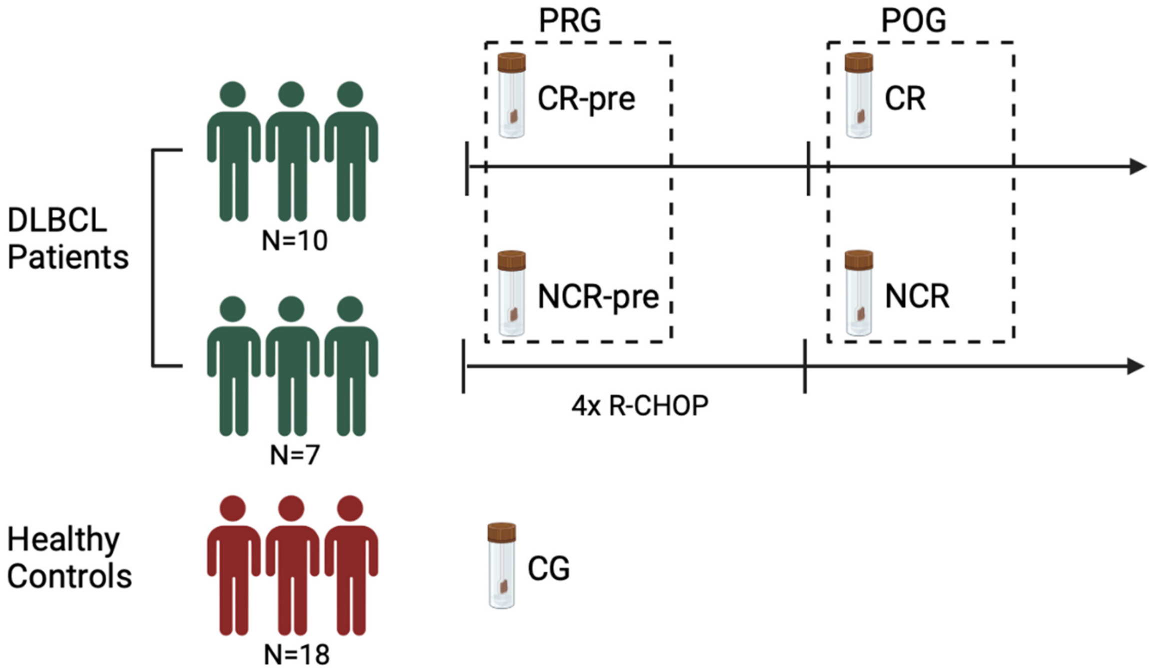

Supplementary Figure S1. Schematic outline of the study design.

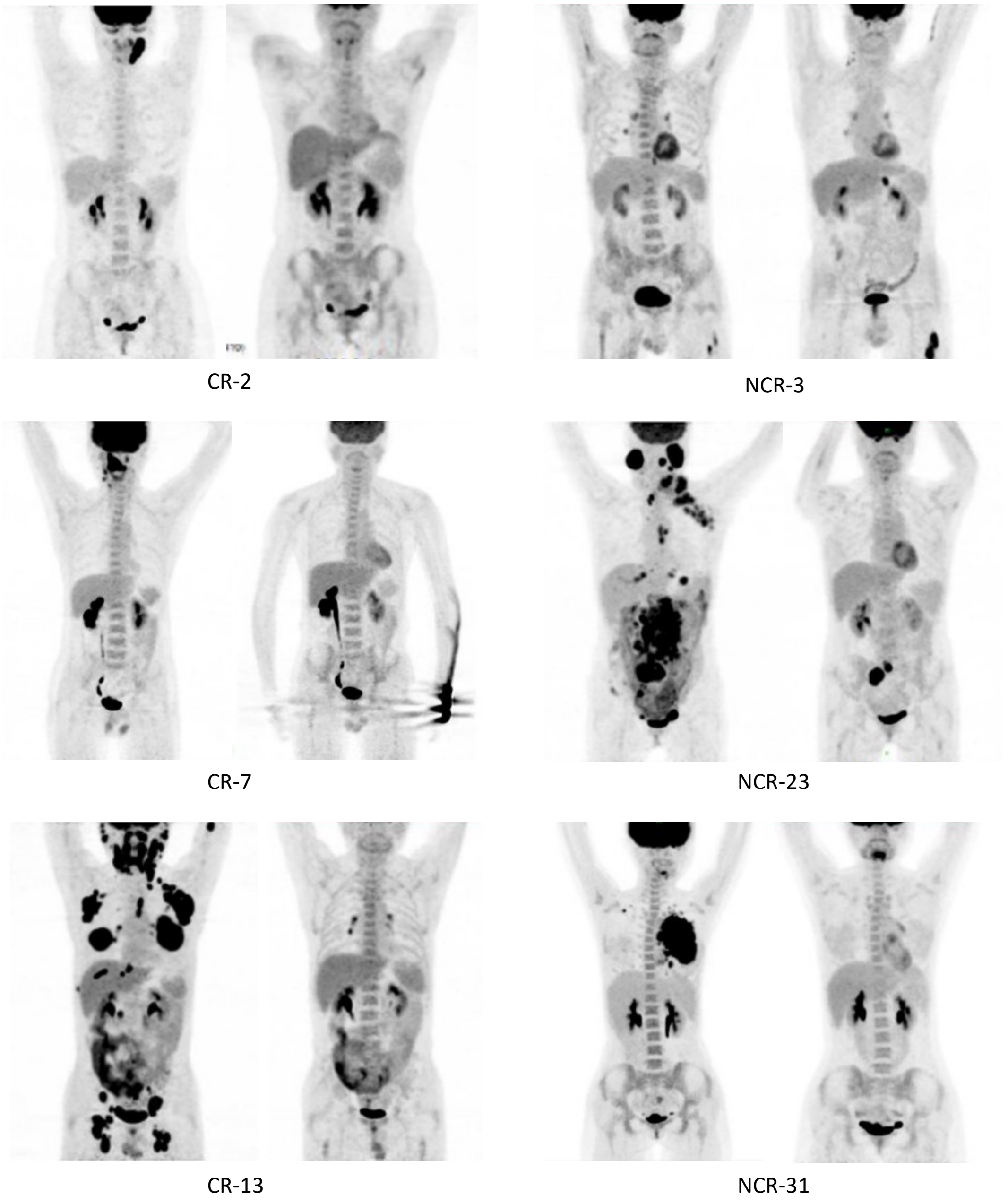

Supplementary Figure S2. PET/CT images of CR/NCR patients. For each patient, left: pre-treatment, right: post-treatment (4x R-CHOP)

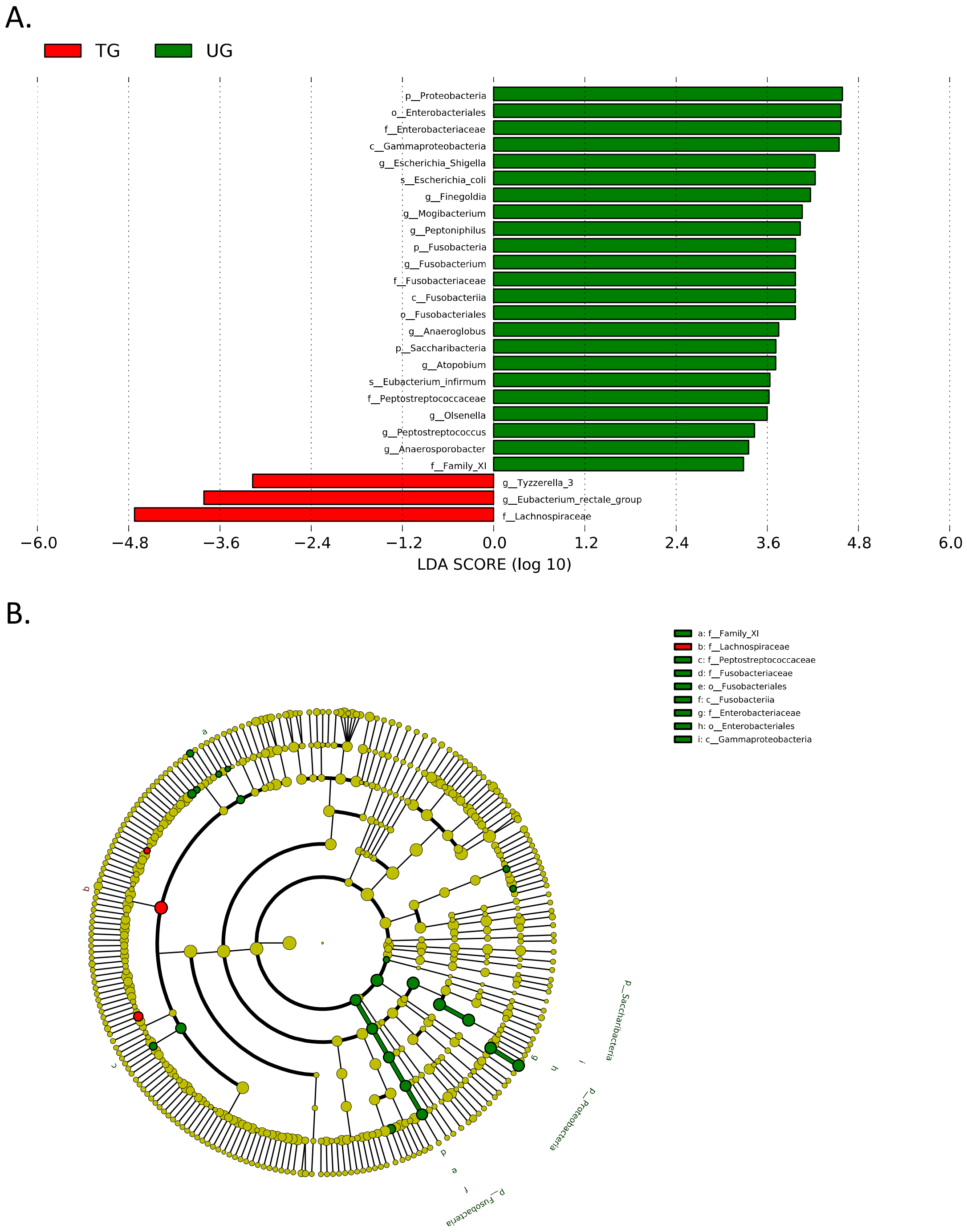

Supplementary Figure S3. GMB composition of untreated patients, and treated patients. (UG: untreated patients, TG: treated patients.) A: LEfSe analysis of taxa abundance, UG vs. TG. B: Cladogram, UG vs. TG.

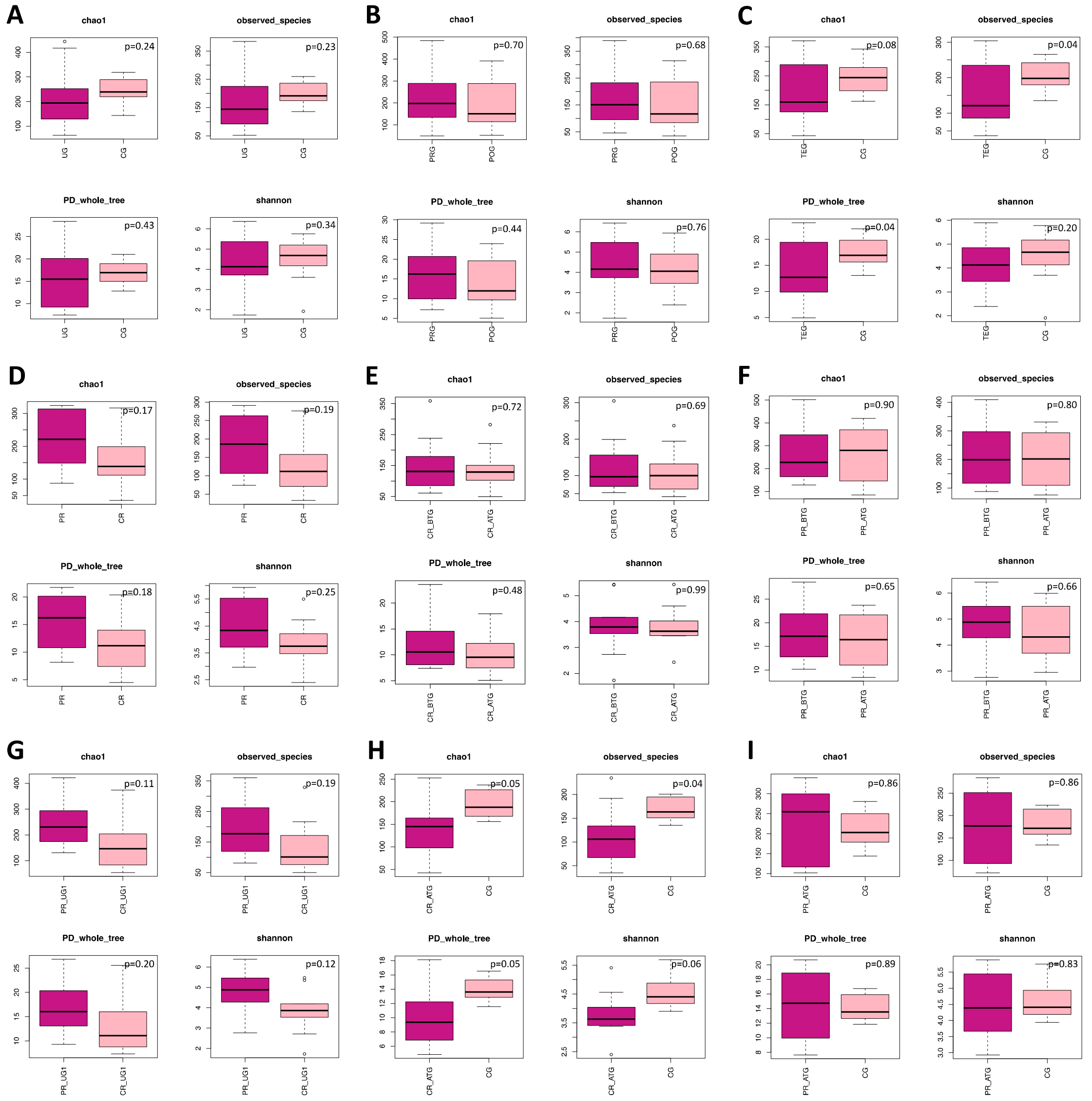

Supplementary Figure S4. A-I: The α-diversity analysis of each pair of groups. The p-values by Turkey-group-test were indicated in the figures.

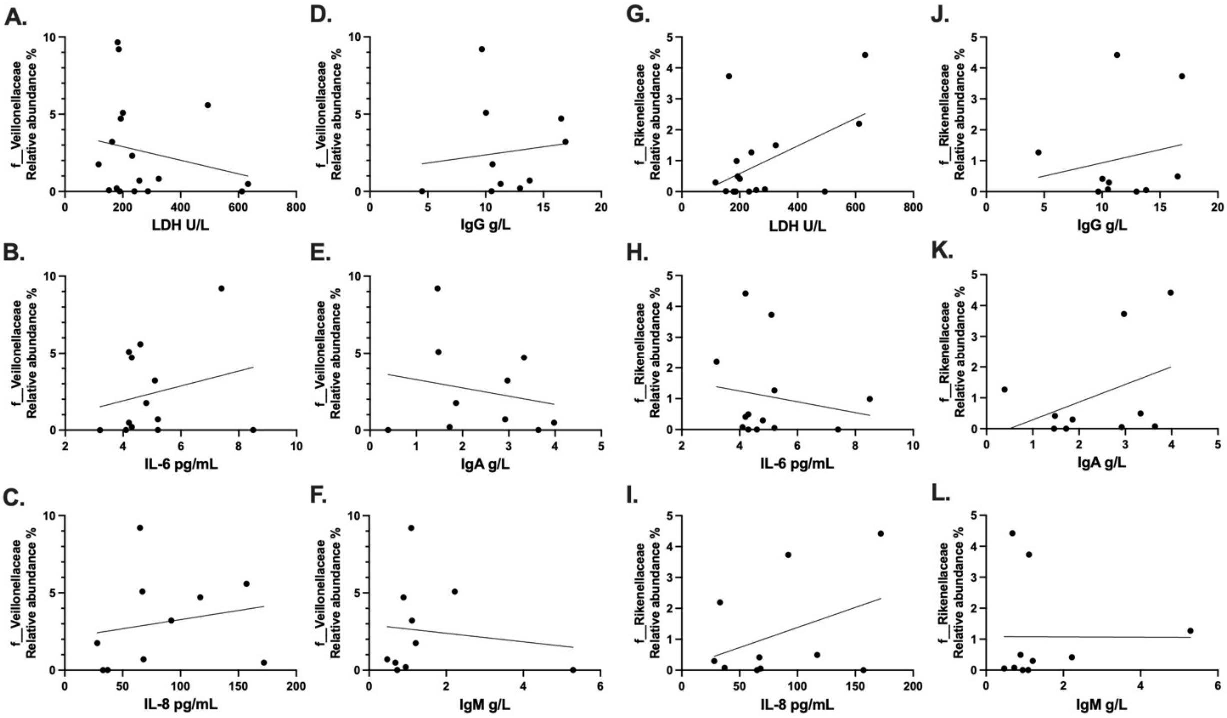

Supplementary Figure S5. A-F: The correlations of serum inflammation markers/immunology markers (LDH, IL-6, IL-8, IgG, IgA, IgM) and relative abundance of *Veillonellaceae*. G-L: The correlations of serum inflammation markers/immunology markers (LDH, IL-6, IL-8, IgG, IgA, IgM) and relative abundance of *Rikenellaceae*.

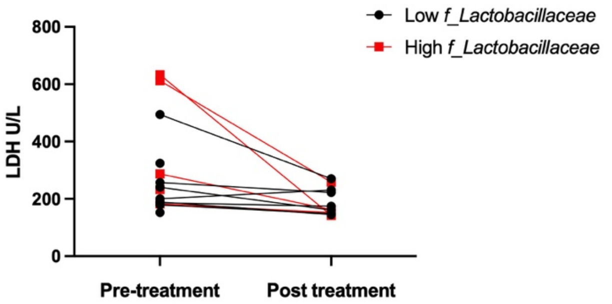

Supplementary Figure S6. The serum LDH level before and after treatment. Patients with high *Lactobacillaceae* abundance (post-treatment) was labeled red color.

1.2 Supplementary Tables

Supplementary Table S1. Virulence factors of *E.coli* in PRG vs. CG

Virulence factors	Gene	p value	Function
**Adhesins**	*fimH*	0.036	d-Mannose-specific adhesin
	bfpA	0.057	Type IV bundle-forming pili
	focG	0.086	Pilus tip molecule
	fliC	0.100	Flagellin variant
	aidA	0.317	Adhesin involved in diffuse adherence
	papC	0.346	Pilus assembly
	fasA	0.584	F6 fimbrial adhesin
	papG	0.681	Gal(1-4)Gal-specific pilus tip adhesin molecule
	papA	0.774	Major structural subunit of pilus
	papEF	0.895	Minor tip pilins
**Capsule synthesis**	*rfc*	0.348	O4 lipopolysaccharide synthesis
**Toxins**	*vat*	0.033	vacuolating toxin
	astA	0.052	Enteroaggregative E.coli toxin
	hlyD	0.057	Hemolysin
	hlyA	0.287	Hemolysin
	east1	0.348	Heat-stable enterotoxin
	clbB/clbN	0.348	Colibactin
	cvaC	0.401	Colicin V
	sat	0.695	Secreted autotransporter toxin
**Siderophores**	*fyuA*	0.032	yersiniabactin receptor
	iroN	0.057	Novel catecholate siderophore
**Additional virulence genes**	*malX*	0.005	Pathogenicity island marker
	traT	0.022	Serum survival
	usp	0.022	Uropathogen-specific protein
	ompT	0.339	Outer membrane protease
	PAI	0.844	Pathogenicity-associated island

Supplementary Table S2. Exopolysaccharides synthesis pathway in *Lactobacillus* in CR vs. NCR

EPS Pathway	Gene	p value	Function
**Protease**	*prtD*	0.103	Protease
	pepN	0.560	Peptidase
**Regulation**	*lytR/epsA*	0.711	Transcriptional regulation of EPS synthesis
**Synthesis**	*epsB*	0.175	Tyrosine kinase modulator
	epsC	0.257	Tyrosine kinase
	epsD	0.870	Phosphotyrosine phosphatase
	epsE	0.158	Priming glycosyltransferase
	epsF	0.036	Unknown
	wzx	0.168	Flippase
	wzy	0.087	Polysaccharide polymerase
**Export**	*epsG*	0.543	Putative membrane protein
	dppA	0.063	Transporter protein
